# Supplementary material for: Tumour-wide RNA splicing aberrations generate actionable public neoantigens
Source: Nature. 2025 Feb 19;639(8054):463–73. doi: 10.1038/s41586-024-08552-0 (PMC11903331; doi:10.1038/s41586-024-08552-0)
Supplement: Supplementary file 2 — Reporting Summary [file 41586_2024_8552_MOESM2_ESM.pdf]

nature portfolio

Corresponding author(s): Hideho Okada, Joseph Costello, Christopher Klebanoff

Last updated by author(s): 06/21/2024

Reporting Summary

Nature Portfolio wishes to improve the reproducibility of the work that we publish. This form provides structure for consistency and transparency in reporting. For further information on Nature Portfolio policies, see our Editorial Policies and the Editorial Policy Checklist.

Statistics

For all statistical analyses, confirm that the following items are present in the figure legend, table legend, main text, or Methods section.

n/a	Confirmed	
☐	☒	The exact sample size (*n*) for each experimental group/condition, given as a discrete number and unit of measurement
☒	☐	A statement on whether measurements were taken from distinct samples or whether the same sample was measured repeatedly
☐	☒	The statistical test(s) used AND whether they are one- or two-sided<br>*Only common tests should be described solely by name; describe more complex techniques in the Methods section.*
☒	☐	A description of all covariates tested
☒	☐	A description of any assumptions or corrections, such as tests of normality and adjustment for multiple comparisons
☐	☒	A full description of the statistical parameters including central tendency (e.g. means) or other basic estimates (e.g. regression coefficient) AND variation (e.g. standard deviation) or associated estimates of uncertainty (e.g. confidence intervals)
☐	☒	For null hypothesis testing, the test statistic (e.g. *F*, *t*, *r*) with confidence intervals, effect sizes, degrees of freedom and *P* value noted<br>*Give P values as exact values whenever suitable.*
☒	☐	For Bayesian analysis, information on the choice of priors and Markov chain Monte Carlo settings
☒	☐	For hierarchical and complex designs, identification of the appropriate level for tests and full reporting of outcomes
☐	☒	Estimates of effect sizes (e.g. Cohen's *d*, Pearson's *r*), indicating how they were calculated

Our web collection on statistics for biologists contains articles on many of the points above.

Software and code

Policy information about availability of computer code

Data collection	Softwares utilized for data collection are detailed below. These softwares include Picard and STAR aligner for RNA-sequencing analysis, MHCFlurry 2.0 and HLAthena for peptide processing and presentation prediction, Cell Ranger for single-cell RNA-sequencing analysis, and MaxQuant for mass spectrometry peptide detection.
Data analysis	Analysis of neojunction expression within multi-region samples across all cancer types were conducted with our neojunction prediction pipeline if the FASTQ file is available. If RNA-sequencing data is only available in BAM format, the sequencing file is converted into FASTQ format utilizing the Picard software (version 2.7.7a). All downloaded RNA-sequencing data sets were individually aligned using a STAR aligner-based processing pipeline (version 2.7.7a). For public cancer-specific splicing event counting, we designed a custom R script that detected and quantified non-annotated, cancer-specific splicing events found across each corresponding patient cohort. Splicing events detected in the GRCh37.87 GTF sj.out.tab (GENCODE v33) file were removed to define non-annotated splicing junctions. A library of all cancer-specific peptides were selected by removing those detectable in normal tissue peptide isoforms in a reference human proteome dataset (UniProt Proteome ID #UP000005640). All cancer-specific peptides with their upstream and downstream flanking sequences (maximum flanking length of 30 amino acids) were independently analyzed and ranked by MHCFlurry 2.0 (v2.1.3) and HLAthena MSiC (v1.0.0). HLA-I binding affinity was assessed against HLA-A*01:01, HLA-A*02:01, HLA-A*03:01, HLA-A*11:01, and HLA-A*24:02 in both cases. In the HLAthena evaluation of antigen binding and presentation to the corresponding HLA haplotypes, peptides were assigned to alleles by rank with a threshold of 0.1. Context of up to 30 flanking amino acids on both N and C terminus were utilized with aggregation by peptide and no log-transformed expression. Baseline MHCFlurry 2.0 models with both peptide:MHC-I binding affinity (BA) predictor and antigen processing (AP) predictor was used. Cell Ranger 7.0.0 (10x Genomics Cloud Analysis) was used to pre-process raw single-cell RNA sequencing and identifying V(D)J clonotypes. Differential gene expression of TCGA, GTEx, and UCSF GBM/LGG RNA-sequencing was performed and quantified using DESeq2. Visualizations of protein folding following splicing aberrations were generated using AlphaFold2. Protein detection from mass spectrometry data was performed utilizing MaxQuant (v1.6.17.0). Flow analysis was performed using FlowJo (v10.9.0).

Custom codes used for analysis are available through the following GitHub link: https://github.com/dakwok/SSNIP

For manuscripts utilizing custom algorithms or software that are central to the research but not yet described in published literature, software must be made available to editors and reviewers. We strongly encourage code deposition in a community repository (e.g. GitHub). See the Nature Portfolio guidelines for submitting code & software for further information.

Data

Policy information about availability of data

All manuscripts must include a data availability statement. This statement should provide the following information, where applicable:

- Accession codes, unique identifiers, or web links for publicly available datasets
- A description of any restrictions on data availability
- For clinical datasets or third party data, please ensure that the statement adheres to our policy

Spatially-mapped glioma biopsy RNA-seq datasets are deposited in the European Genome-Phenome Archive under accession numbers EGAS00001007986, EGAS00001006785, EGAD00001005221/2, EGAD00001009496/7. Spatially-mapped biopsy RNA-seq data for other tumor types were retrieved from their corresponding publications: Through the NIH SRA at https://www.ncbi.nlm.nih.gov/sra, RNA-seq data can be accessed with accession ID PRJNA579899 for Ku et al. 2018, SRP066596 for Joung et al. 2016. Through the National Omics Data Encyclopedia (NODE), RNA-seq data can be accessed with accession code OEP002956 for Yang et al. 2022. Through EGA, RNA-seq data can be accessed with accession code EGAD00001009042 for Jeon et al. 2023, EGAS00001003813 for Zhai et al. 2023, EGAS00001005328 for Meiller et al. 2021. TRACERx data was requested and received from the Cancer Research UK & University College London Cancer Trials Centre. Glioma MS data was retrieved from the CPTAC Consortium as well as the Proteomics Identifications Database (PRIDE). PRIDE accession code for Bader et al. 2021 is PXD024427. Proteomic data from Wong et al. 2022 was retrieved from their supplementary files.

Research involving human participants, their data, or biological material

Policy information about studies with human participants or human data. See also policy information about sex, gender (identity/presentation), and sexual orientation and race, ethnicity and racism.

Reporting on sex and gender	N/A
Reporting on race, ethnicity, or other socially relevant groupings	N/A
Population characteristics	N/A
Recruitment	N/A
Ethics oversight	N/A

Note that full information on the approval of the study protocol must also be provided in the manuscript.

Field-specific reporting

Please select the one below that is the best fit for your research. If you are not sure, read the appropriate sections before making your selection.

☒ Life sciences ☐ Behavioural & social sciences ☐ Ecological, evolutionary & environmental sciences

For a reference copy of the document with all sections, see nature.com/documents/nr-reporting-summary-flat.pdf

Life sciences study design

All studies must disclose on these points even when the disclosure is negative.

Sample size	Experimental sample size was determined based on significance, expectations of variability for specific experiment types, and by feasibility in large scale undertakings. Biological and technical triplication was carried out for all experiments to ensure statistical robustness. No formal sample size calculation was performed for this study. The sample sizes were chosen based on practical considerations, including the typical variability observed in similar experiments and the feasibility of generating or analyzing samples within the constraints of available resources, such as time, budget, and experimental materials.
Data exclusions	No data were excluded.
Replication	Data were reproduced at technical, biological, and experimental levels where relevant replication information is provided. Repeats agreed with one another, and all findings described in this manuscript were confirmed in at least two independent experimental repeats, demonstrating reliable reproducibility. To ensure robustness, flow analysis experiments were performed in most cases using two independent target antibodies.
Randomization	Randomization was not relevant to our study because all experiments were conducted in a controlled benchwork setting where external sources of variability were minimized. The experimental procedures involved standardized reagents, equipment, and protocols, ensuring

uniform conditions across all replicates.

Blinding | Blinding is not applicable to this study.

Reporting for specific materials, systems and methods

We require information from authors about some types of materials, experimental systems and methods used in many studies. Here, indicate whether each material, system or method listed is relevant to your study. If you are not sure if a list item applies to your research, read the appropriate section before selecting a response.

Materials & experimental systems

n/a	Involved in the study
☐	☒ Antibodies
☐	☒ Eukaryotic cell lines
☒	☐ Palaeontology and archaeology
☒	☐ Animals and other organisms
☒	☐ Clinical data
☒	☐ Dual use research of concern
☒	☐ Plants

Methods

n/a	Involved in the study
☒	☐ ChIP-seq
☐	☒ Flow cytometry
☒	☐ MRI-based neuroimaging

Antibodies

Antibodies used

APC anti-human HLA-A2 Antibody [clone BB7.2] Invitrogen Ref #17-9876-42
APC anti-human CD8a Antibody [clone SK1] BioLegend Cat #344721
APC anti-human CD107a Antibody (LAMP-1) [clone H4A3] BioLegend Cat #328620
APC anti-human TCR α/β Antibody [clone IP26] BioLegend Cat #306718
APC/Cyanine7 anti-human TCR α/β [clone IP26] BioLegend Cat #306728
APC/Cyanine7 anti-human CD3 Antibody [SK7] BioLegend Cat #344818
FITC anti-human HLA-A3 Antibody [clone BB7.2] BioLegend Cat #343304
FITC anti-human CD3 Antibody [clone UCHT1] BioLegend Cat #300440
PE anti-human TCR α/β Antibody [clone EP26] BioLegend Cat # 306708
PE anti-human HLA-A2 Antibody [clone BB7.2] BioLegend Cat #343306
PE anti-mouse TCR β chain Antibody [clone H57-597] BioLegend Cat #109208
PE anti-human CD137 (4-1BB) Antibody [clone 4B4-1] BioLegend Cat #309804
PE anti-human CD3 Antibody [clone HIT3a] BioLegend Cat #300308
Zombie GreenTM Fixable Viability Kit BioLegend Cat #423112
InVivoMAb anti-human MHC Class I Antibody W6/32, Bio X Cell, Cat. #BE0079
Unless otherwise stated, the concentration of antibody used is the one recommended by the manufacturer.
Lot number information was not recorded.

Validation

All antibodies used in this study are commercially available and have been validated by the manufacturer or in previous reports. In this specific study, the antibodies were not validated as we used trusted commercial sources and followed the manufacturer's recommended protocols and applications:

APC anti-human HLA-A2 Antibody [clone BB7.2] Invitrogen Ref #17-9876-42 (https://www.thermofisher.com/antibody/product/HLA-A2-Antibody-clone-BB7-2-Monoclonal/17-9876-42)

APC anti-human CD8a Antibody [clone SK1] BioLegend Cat #344721 (https://www.biolegend.com/en-ie/products/apc-anti-human-cd8-antibody-6531)

APC anti-human CD107a Antibody (LAMP-1) [clone H4A3] BioLegend Cat #328620 (https://www.biolegend.com/de-at/products/apc-anti-human-cd107a-lamp-1-antibody-5428)

APC anti-human TCR α/β Antibody [clone IP26] BioLegend Cat #306718 (https://www.biolegend.com/fr-ch/products/apc-anti-human-tcr-alpha-beta-antibody-6704)

APC/Cyanine7 anti-human TCR α/β [clone IP26] BioLegend Cat #306728 (https://www.biolegend.com/en-gb/products/apc-cyanine7-anti-human-tcr-alpha-beta-antibody-12516)

APC/Cyanine7 anti-human CD3 Antibody [SK7] BioLegend Cat #344818 (https://www.biolegend.com/en-gb/products/apc-cyanine7-anti-human-cd3-antibody-6940?GroupID=BLG5900)

FITC anti-human HLA-A3 Antibody [clone BB7.2] BioLegend Cat #343304 (https://www.biolegend.com/nl-nl/products/fitc-anti-human-hla-a2-antibody-6018?GroupID=BLG7410)

FITC anti-human CD3 Antibody [clone UCHT1] BioLegend Cat #300440 (https://www.biolegend.com/en-gb/clone-search/fitc-anti-human-cd3-antibody-863?GroupID=BLG5900)

PE anti-human TCR α/β Antibody [clone EP26] BioLegend Cat # 306708 (https://www.biolegend.com/en-ie/products/pe-anti-human-

alpha-beta-t-cell-receptor-antibody-773?GroupID=GROUP28)

PE anti-human HLA-A2 Antibody [clone BB7.2] BioLegend Cat #343306 (https://www.biolegend.com/fr-fr/products/pe-anti-human-hla-a2-antibody-6175)

PE anti-mouse TCR β chain Antibody [clone H57-597] BioLegend Cat #109208 (https://www.biolegend.com/de-at/products/pe-anti-mouse-tcr-beta-chain-antibody-272)

PE anti-human CD137 (4-1BB) Antibody [clone 4B4-1] BioLegend Cat #309804 (https://www.biolegend.com/en-gb/products/pe-anti-human-cd137-4-1bb-antibody-1510?GroupID=BLG2203)

PE anti-human CD3 Antibody [clone HIT3a] BioLegend Cat #300308 (https://www.biolegend.com/fr-lu/products/pe-anti-human-cd3-antibody-753)

Zombie GreenTM Fixable Viability Kit BioLegend Cat #423112 (https://www.biolegend.com/fr-ch/products/zombie-green-fixable-viability-kit-9340)

InVivoMAb anti-human MHC Class I Antibody W6/32, Bio X Cell, Cat. #BE0079 (https://bioxcell.com/invivomab-anti-human-mhc-class-i-hla-a-hla-b-hla-c-be0079)

Eukaryotic cell lines

Policy information about cell lines and Sex and Gender in Research

Cell line source(s)	GBM cell lines were obtained from the Mayo Clinic Brain Tumor Patient Derived Xenograft National Resource (Vaubel et al., 2020). LGG cell lines were obtained from an in-house consortium (Jones et al., 2020). Triple-reporter Jurkat76 cells were obtained by MTA through the Robert Prins Lab at UCLA. COS7 and T2 cells were obtained through ATCC. Pan-cancer cell lines were obtained through a various sources as referenced in Stevers et al., 2023.
Authentication	Cell lines were authenticated by short tandem repeat (STR) analysis at the University of California Berkeley Sequencing Facility and by the commercial providers where applicable.
Mycoplasma contamination	Cell lines were confirmed to be Mycoplasma free by PCR using previously published methods.
Commonly misidentified lines (See ICLAC register)	No commonly misidentified cell lines were used in this study.

Plants

Seed stocks	N/A
Novel plant genotypes	N/A
Authentication	N/A

Flow Cytometry

Plots

Confirm that:

- [x] The axis labels state the marker and fluorochrome used (e.g. CD4-FITC).
- [x] The axis scales are clearly visible. Include numbers along axes only for bottom left plot of group (a 'group' is an analysis of identical markers).
- [x] All plots are contour plots with outliers or pseudocolor plots.
- [x] A numerical value for number of cells or percentage (with statistics) is provided.

Methodology

Sample preparation	PBMCs, T-cells (CD8+ T-cell and Jurkat76 cell lines), APCs, and other cell lines were collected, washed with FACS buffer, and stained with the corresponding antibodies as per manufacturer's instructions. Cells were washed twice with FACS buffer prior to analysis.

Instrument	Cells were analyzed at the UCSF Helen Diller Comprehensive Cancer Center using the ATTUNE2 or ATTUNE NxT Flow Cytometer. Flow sorting was performed on the BD Biosciences FACSAria Flow Cytometer.
Software	Data was collected using the BD Biosciences FACSDiva software v6.1.3 or the Attune Cytometric software. Analysis was performed using FlowJo v10.7.2.
Cell population abundance	At least 1e6 cells were collected for analysis and 5e4 cells were analyzed.
Gating strategy	Using the FSC/SSC gating, debris was removed, and the single alive cells were gated with the additional live/dead staining. Each population was gated based on the surface or intracellular markers as described in the manuscript. Details are provided in the main text and the extended data.

☒ Tick this box to confirm that a figure exemplifying the gating strategy is provided in the Supplementary Information.
